# Identification of airborne microbiota in selected areas in a health-care setting in South Africa

**DOI:** 10.1186/1471-2180-14-100

**Published:** 2014-04-22

**Authors:** Gaofetoge Setlhare, Ntsoaki Malebo, Karabo Shale, Ryk Lues

**Affiliations:** 1 Department of Life Sciences, Unit for Applied Food Science and –Biotechnology, Central University of Technology, Free State, Private Bag X 20539, Bloemfontein 9300, South Africa

**Keywords:** Air quality, Bio-aerosols, Health-care setting, MALDI-TOF MS

## Abstract

**Background:**

The role of bio-aerosols in the spread of disease and spoilage of food has been described in numerous studies; nevertheless this information at South African hospitals is limited. Attributable to their size, bio-aerosols may be suspended in the air for long periods placing patients at risk of infection and possibly settling on surfaces resulting in food contamination. The aim of the study is to assess the microbial composition of the air in the kitchen and selected wards at a typical district hospital in South Africa. Air samples were collected using the settle plates and an SAS Super 90 air sampler by impaction on agar. These microbial samples were quantified and identified using Matrix Assisted Laser Desorption/Ionization Time of Flight Mass Spectrometry (MALDI-TOF MS) and Analytic Profile Index (API).

**Results:**

Microbial counts were found to be higher in the fourth (≤6.0 × 10^1^ cfu/m^-3^) sampling rounds when compared to the first (≥2 cfu/m^-3^), second (≤3.0 × 10^1^ cfu/m^-3^) and third (≤1.5 × 10^1^ cfu/m^-3^) sampling rounds. Genera identified included *Bacillus*, *Kocuria*, *Staphylococcus*, *Arthrobacter*, *Candida, Aureobasidium, Penicillium* and *Phoma* amongst others*.* The presence of these pathogens is of concern, attributable to their ability to cause diseases in humans especially in those with suppressed host immunity defenses. Furthermore, fungal genera identified (e.g. *Candida*) in this study are also known to cause food spoilage and fungal infections in patients.

**Conclusion:**

Results from this study indicate the importance of air quality monitoring in health-care settings to prevent possible hospital-acquired infections and contamination of hospital surfaces including food contact surfaces by airborne contaminants.

## Background

Bio-aerosols are airborne particles that originate from living microorganisms such as bacteria, fungi, and viruses generally found as part of the patient’s endogenous flora. Their components have negative effects especially on the health of immuno-compromised people [1]. The infectious aerosols are small and may remain suspended and viable in the air stream over long periods of time. Attributable to the above-mentioned facts, the risk of airborne infections especially in hospitals and other health-care settings can be high as there can be confined spaces in which aerosols may build up to infectious levels [1]. The buildup of infectious aerosols exacerbates healthcare challenges in developing countries as the role of bio-aerosols in hospital-acquired infections (HAIs) has been recognized; studies have uncovered increasing evidence regarding the spread of disease via the aerial route [2]. Consequently, the presence of bio-aerosols in health-care settings needs to be monitored and controlled to limit their dispersal.

The presence of bio-aerosols in hospitals, especially in different wards, may be attributed to infected patients who transmit these contaminants via the aerial route [2]. Transmission occurs when microbial pathogens are released from an infected patient to vulnerable individuals through activities such as coughing, sneezing and talking [3]. Recent studies have demonstrated that challenging pathogens such as methicillin-resistant *Staphylococcus aureus* (MRSA) may spread via the aerial route, which can lead to an increase in hospital-acquired infections and the spread of antibiotic resistant genes [4]. Other possible sources of bio-aerosols in hospital may be clothes or other personal items belonging to patients [4].

In the health-care environment, kitchens play a critical role in food safety; the safety and the quality of food served in hospitals depends on the kitchen design and storage conditions, as well as on the food preparation practices of food handlers [5]. Numerous studies have revealed that food handlers may also contribute in the distribution of airborne microbial contaminants through activities such as coughing, sneezing and talking. Food handlers, however, also play a key role in the prevention of food contamination during food production, handling and distribution, a point that has also been widely highlighted [5]. Interestingly, bacterial contamination in the kitchen may also be attributable to bacterial loads on paper towels and hand-towels which when used release bacteria including spores, increasing airborne microbial loads and possibly settling on food contact surfaces [6]. Bacteria mainly isolated from paper towels are the toxin-producing *Bacillus* that has been implicated in cases of food poisoning. As a result, kitchens are believed to be other possible contributing factors in the spread of food-borne and infectious diseases including airborne microbial contaminants [6].

The presence of airborne foodborne pathogens such as *B. cereus* and *S. aureus* that have been implicated in several HAI cases is of great concern in health-care settings. This does not exclude other foodborne hospital-acquired pathogens such *Campylobacter jejuni*, *Clostridium perfringens*, *Klebsiella* spp., *Salmonella* spp., *Pseudomonas aeruginosa*, and *Escherichia coli* that can also be transmitted via the aerial route [7,8]. In addition, the presence of fungi in the health-care environment has also been implicated in numerous HAI cases. Although aerosolised fungi have been known mainly to cause food spoilage, literature has shown that airborne fungi may result in infectious diseases such as aspergilloses, candidoses, coccidioidomycosis, cryptococcosis, histoplasmosis, mycetomas and paracoccidioidomycosis [9].

Lack of reports especially in South Africa regarding the composition and quantity of airborne microbial contaminants especially in health-care settings is a concern attributable to the increasing risk associated with contracting HAI via the aerial route [10,11]. The gradual development of drug resistance and sudden emergence and re-emergence of infectious disease around the world including infection control challenges at South African hospitals highlights a need to improve existing health systems. Although HAIs are commonly associated with person-to-person contact, cases of transmission via the aerosol route have been reported in various studies [4,12]. There is enough evidence that suggests that the presence of bio-aerosols in hospitals is a threat to people with poor immune systems, particularly in South Africa which has high numbers of patients with HIV/AIDS and TB amongst other diseases [5]. The aims of this study were to quantify aerosolised microbes in food preparation areas and selected wards using active and passive sampling methods. Consequently Analytic Profile Index (API) and a Matrix-Assisted Laser Desorption/Ionization Time of Flight Mass Spectrometry (MALDI-TOF MS) shall be used to identify isolated organisms.

## Methods

### Sampling sites

The study was conducted at a district hospital in the Free State province. The hospital is amongst the oldest government hospitals built. Air samples were taken from the following sites: the entire kitchen area (KA), male ward corridor (MWC), male ward room 3 (MWR3), male ward room 4 (MWR4), male ward room 5 (MWR5), male ward TB room (MWTB), female ward corridor (FWC), female ward room 40 (FWR40), female ward preparation room (FWPR) and diabetic female ward (DFW). In each setting, air samples were collected twice over four rounds in duplicate at different time periods (between 10:00 – 12:00) during preparation of food. The samples were kept on ice during transportation to the laboratory and analysed without delay on arrival.

### Air sampling

Two methods (passive and active air sampling) were used to monitor microbial activity in the air at the hospital. Passive sampling was selected because it provides information about the long-term contamination of the studied environmental compartment. Additionally, this method can be used to predict possible contamination of surfaces as it allows measurement of settling microorganisms. Active air sampling is recommended when the concentration of microorganisms is not high [13]. This method can also be used to obtain information on the concentration of inhalable airborne particles in indoor environments. In the current study, both methods were used because this is the first time a study on air monitoring is conducted at the selected hospital.

#### *Active sampling*

Air samples were collected 1.5 meters above the floor on Plate Count Agar (PCA) and Potato Dextrose Agar (PDA) plates using the SAS Super 90 air sampler (Rodac Nunc, Denmark). The air sampler was calibrated at an airflow rate of 0.03 m^3.^min^-1^ and detachable parts were autoclaved before use and sterilized with 70% ethanol between sampling runs [14]. PCA and PDA were used (Merck, SA) for the isolation of total viable aerobic counts and total fungi respectively. Samples were then placed in a cooler box and transported to the laboratory for further analysis. The plates were incubated at 25°C for fungi and 37°C for bacteria for 24 to 72 hours. Sampled air volume concentrations were calculated using the positive-hole conversion table provided by the manufacturer. Colonies were specified and expressed as colony-forming units per cubic meter of air (cfu/m^-3^).

#### *Passive sampling*

Moreover, the settle plate method was used for measuring the rate of deposition of large particles from air [13,15]. The current method was used to determine the *Index of Microbial Air Contamination* (IMA). According to literature [15] the index corresponds to the number of colony forming units (CFU) on a Petri dish with a diameter of 90 mm placed for 1 hour, 1 m above the floor about 1 m away from obstacles and walls. In the current study, IMA plates were placed according to the method of Napoli et al. [15] at the following sites: the kitchen area (KA), male ward corridor (MWC), male ward room 3 (MWR3), male ward room 4 (MWR4), male ward room 5 (MWR5), male ward TB room (MWTB), female ward corridor (FWC), female ward room 40 (FWR40), female ward preparation room (FWPR) and diabetic female ward (DFW). In each setting, air samples were collected twice over four rounds in duplicate at different time periods (between 10:00 – 12:00) during preparation of food. The samples were kept on ice during transportation to the laboratory and analyzed without delay on arrival.

### Microbial sample preparation for API

For sample collection and preparation, the microorganisms to be identified were first isolated on a selective culture medium (Baird Parker Agar (Oxoid) for *Staphylococcus*; *Bacillus cereus* Selective Agar (Oxoid) for *Bacillus*; Chromocult agar (Merck, South Africa) for coliforms) according to standard microbiological techniques. After sample preparation, colonies (from the selective media agar plates) were emulsified into the API Medium to achieve a homogeneous bacterial suspension of a 0.5 McFarland standard. The suspension was used immediately after preparation. A sterile pipette was used to distribute the bacterial suspension into the tubes. After inoculation of strips, the incubation box was immediately closed and incubated at 36°C ± 2°C for 18–24 hours. The strips were read after the stipulated incubation period (24 hours, 48 hours and/or 72 hours, depending on the microorganism and the type of reaction studied). For the interpretation of results, a numerical profile was used and for identifying bacterial species, a database (V4.0) was performed with the analytical profile index by looking up the numerical profile in the list of profiles or with the identification software by entering the 7-digit numerical profile manually [16].

### Microbial sample preparation for MALDI-TOF MS

The MALDI Biotyper uses Matrix Assisted Laser Desorption/Ionization Time of Flight Mass Spectrometry (MALDI-TOF MS) for microbial identification. The MALDI-TOF MS Microflex mass spectrometer (Bruker Daltonik, Bremen, Germany) FlexControl software (version 3.0) measures highly abundant proteins that are found in all microorganisms. The characteristic patterns of these highly abundant proteins are used to reliably and accurately identify a particular microorganism by matching the respective pattern with an extensive open database to determine the identity of the microorganism down to the species level (Bruker). For identification of colonies using the MALDI-TOF MS; direct placing or placing on a steel target following extraction was done (according to the manufacturer’s instructions). Briefly, single colony from each plate was picked up and smeared as a thin film directly on a MALDI steel target. Microorganisms that could not be identified directly by MALDI-TOF MS underwent extraction and were retested. Pure colonies were transferred to a 1.5 ml tube (Eppendorf, Germany) mixed thoroughly in 300 μl of distilled water. Nine hundred micro liters (900 μl) of absolute ethanol were added, the mixture was centrifuged at 15,500 *g* for 2 min, and the supernatant was discarded. The pellet was air-dried at room temperature. Subsequently, 50 μl of formic acid (70% v/v) was added to the pellet and mixed thoroughly before the addition of 50 μl of acetonitrile. The mixture was centrifuged again at 15,500 *g* for 2 min. One microliter of the supernatant was placed onto a spot of the steel target and air-dried at room temperature. Following this, 1 μl of matrix solution (20 mg/ml 3, 5-dimethoxy-4-hydroxycinnamic acid in acetonitrile (ACN): purified water: trifluoroacetic acid (TFA) (50:50:0.1)) was used to overlay the smeared colonies on the steel target. The steel target was air-dried for 10 minutes and placed in the MALDI Biotyper for analysis. Measurements were done using a Microflex Mass Spectrometer (Bruker Daltonik, Bremen, Germany) with FlexControl software (version 3.0). Spectra were recorded in the positive linear mode (laser frequency, 20 Hz; ion source 1voltage, 20 kV; ion source 2 voltage, 18.4 kV; lens voltage, 9.1 kV; mass range, 2,000 to 20,000 Da). For each spectrum 240 shots in 40-shot steps from different positions of the target spot (automatic mode) were collected and analysed. All colonies reported were above 1.80 score value. Identification of unknown microbes found in the hospital was classified using modified score values proposed by the manufacturer: a score of ≥2 indicated species identification; a score between 1.7 and 1.9 indicated genus identification and a score of <1.7 indicated not reliable identification [17].

## Results and discussion

### Quantification of bacterial airborne contaminants

**Figure 1:**
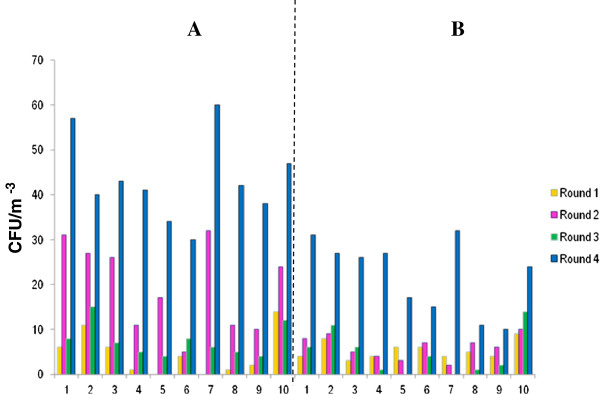
Cultivable airborne bacteria isolated using (A) settling plates and (B) SAS-super 90 in (Kitchen area (1), male ward corridor (2), male ward room 3 (3), male ward room 4 (4), male ward room 5 (5), male ward TB room (6), female ward corridor (7), female ward room 40 (8), female ward preparation room (9) and diabetic female ward (10)).

**Figure 2:**
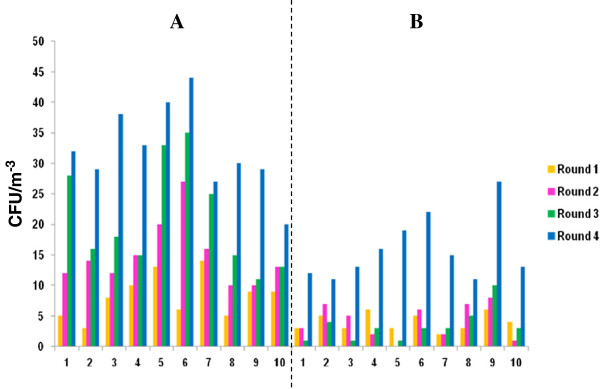
Cultivable airborne fungi isolated using (A) settling plates and (B) SAS-super 90 in (Kitchen area (1), male ward corridor (2), male ward room 3 (3), male ward room 4 (4), male ward room 5 (5), male ward TB room (6), female ward corridor (7), female ward room 40 (8), female ward preparation room (9) and diabetic female ward (10)).

During sampling rounds, bacterial counts obtained using settle plates and SAS-Super 90 in both the kitchen area and wards (male and female) ranged between ≥ 2 cfu/m^-3^ for the first sampling round, ≤ 3.0 × 10^1^ cfu/m^-3^ for the second sampling round, ≤ 1.5 × 10^1^ cfu/m^-3^ for the third sampling round and ≤ 6.0 × 10^1^ cfu/m^-3^ in the fourth sampling round (Figure 1). Generally, counts observed were higher when passive sampling was used in comparison to the active sampling (Figures 1 and 2). The differences observed using both sampling methods were statistically significant for the bacterial samples p = 0.0015 (Figure 1). The results were comparable with results observed elsewhere [15]. In the current study, the fourth sampling round using both sampling methods higher counts were observed when values were compared with those obtained in other sampling rounds (the first, second and third). This was due to increased human activity (e.g. large number of patients, personnel, and visitors occupying the hospital wards within a short period of time) in rooms as well as corridors while in the first three sampling rounds patients were discharged from the hospital thus there was less activity. The current results are similar to results observed in a study conducted in 2012 [15] where human activity resulted in higher total viable counts. Throughout the entire kitchen area (≤5.8 × 10^1^ cfu/m^-3^), male (≤4.3 × 10^1^ cfu/m^-3^) and female wards (≤6.0 × 10^1^ cfu/m^-3^) in the last round demonstrated high microbial levels (Figure 1) using both sampling methods. Airborne contaminants are usually introduced into the air through production of aerosol droplets by humans via coughing, sneezing and talking. Possible sources of bio-aerosols in hospitals are commonly patients, staff and hospital visitors [18] and results in the current study also indicate these as possible sources that may have led to an increase in bio-aerosol counts in the fourth rounds. However, no attempts were made in the current study to correlate air samples with clinical samples or with samples from other hospital occupants, which was a noted limitation in the current study.

The presence of these contaminants in the air may inadvertently introduce pathogenic organisms into the body that at a later stage may cause HAIs [19]. In addition, mainly because of improper food hygiene practices and especially improper cleaning of surfaces, food handlers may be carriers of airborne contaminants that may settle on food preparation areas and be transferred to patients. Poor food hygiene practices remains a serious public health problem [20] as it is known to cause food poisoning especially to vulnerable groups such as the sick, elderly, young children and pregnant women. During the third sampling visit the male ward (Room 4), male ward (Room 5), female ward corridor, female ward prep room and female ward (Room 40) had the lowest bacterial counts. This may be attributable to lack of activity in these rooms since patients were discharged at that time of sampling. Counts obtained in this study were lower (≤6.0 × 10^1^ cfu/m^-3^) when compared with counts (2.54 × 10^2^ cfu/m^-3^) obtained in another study by Qudiesat and co-workers [19], and furthermore, counts in the current study were even lower in comparison to the levels of acceptable microbial population at hospitals. This is the first report on levels of bio-aerosols at this hospital. Even though bacterial counts were low, results indicate biological activity in the air at this hospital that indicates a need for intervention since this is the first report of bioaerosol’ quantification at the hospital under study. Frequent air monitoring is necessary in health-care settings because an increase in microbial counts may place patients as well as staff at high risk of contracting airborne pathogenic microorganisms. Additionally, when the level of microbial activity is known, hospital environmental control procedures can be implemented as an ideal control measure to reduce HAI.

### Quantification of fungal airborne contaminants

In general, fungal counts (Figure 2) obtained using the passive and active method in the kitchen area and the, male and female wards ranged between ≥ 4 cfu/m^-3^, that were isolated during the first sampling round, ≥ 4 cfu/m^-3^ in the second sampling round, ≥ 2 cfu/m^-3^ in the third sampling round, and ≤ 4.5 × 10^1^ cfu/m^-3^ in the fourth sampling round. Again counts obtained using passive sampling were higher than counts obtained with active sampling, the differences observed were statistically significant p = 0.0001 (Figure 2). The current results were contrary to results observed elsewhere [15] where active sampling was reportedly better at collecting fungal species. The differences are possibly due to the sampling environment which was different in the two studies, Napoli et al. [15] collected samples from a controlled environment whereas samples in the current study were from an uncontrolled hospital environment. Generally, counts for bacteria and fungi were similar as indicated in the respective figures (Figures 1 and 2). To determine the exact relationships amongst various microbiota, Spearman’s correlation coefficient and F-Test (two-tailed probability) were used to construct a correlation matrix and significant differences. Microbial counts in the kitchen area and the, male and female wards showed a correlation coefficient between bacteria and fungi to be r^2^ = 0.5 (first sampling rounds), r^2^ = 0.07 (second sampling rounds), r^2^ = -0.01 (third sampling rounds) and r^2^ = -0.3 (fourth sampling rounds) respectively. The first and second sampling rounds showed a fair positive correlation, whereas a negative poor correlation for third and fourth sampling rounds was observed. In addition, no significant difference was observed for bacteria during the first and second four sampling rounds (p = 0.798) additionally no significant difference was observed for fungi during the first and second four sampling rounds (p = 0.981).

The fourth sampling round also showed high fungal counts (Figure 2), approximately 4.5 × 10^1^ cfu/m^-3^; this was high when compared to other sampling rounds (the first, second and third sampling rounds). From the results, possible sources of fungal airborne contaminants increasing microbial levels may be attributable to the high level of human activity observed during the fourth sampling round that resulted to a need to open windows, and possibly to the introduction of outdoor fungi to the indoor areas. Other possible sources include inadequate air filtration systems: insufficient air filtering may provide easy access to the hospital indoor environment for mould spores [5,21]. Additional studies to assess the efficacy of the air filtration systems shall have to be assessed in future. In addition, Pastuszka and colleagues [22] report that surfaces and problems such as painted surfaces, wallpapers, cracks, holes, ceilings and dust may be major sources of fungal contamination causing serious infections to patients. Fungal spores can accumulate in hospital areas when dust enters the patient’s room as a contaminant on the clothing of personnel, such as on aprons or uniforms, or even on the patient’s personal items [22,23]. Even though fungal counts were high, visible fungal growth on walls and ceilings was not observed during sampling.

Throughout sampling, the first, second and third rounds low fungal counts (6 cfu/m^-3^) were observed in the kitchen. This may be because during those sampling rounds some food handlers were absent and the kitchen was not as busy as it was during the fourth sampling round. In general, bacterial levels were found to be higher and more sensitive when compared to fungal levels, in relation to all activities of workers and to the number of people in each ward and corridors. Moreover, the results in this study were found to be similar to results obtained by [24-26]. However, it is also true that fungal counts obtained by Qudiesat et al. [19] were compared to fungal counts obtained in this study and the results quantified showed low counts (≥2 cfu/m^-3^) when correlated to results the other studies (7.3 × 10^1^ cfu/m^-3^). For the identification of unknown bacteria and fungi present in kitchen areas and selected wards, MALDI-TOF MS and API tests were performed.

### Bacterial characterization

In the entire kitchen area (Table 1), *Bacillus cereus* was identified using both MALDI-TOF MS and API. Studies have shown that the source of this Gram-positive bacterium may be paper towels, and interestingly food handlers at the hospital studied used paper towels for cleaning, covering or wrapping food [6]. This current practice is of concern because covering or wrapping food with contaminated paper towels may result in possible infections or food poisoning. Moreover, other possible sources of this bacterium may be dust particles, since *B. cereus* is found in soil and its presence on a dust particle in the air may result in settling on food and food contact surfaces. *B. cereus* can multiply and survive in unfavorable conditions such as very low and also very high temperatures due to its ability to form spores [27], thus ensuring its survival in the kitchen and posing a possible threat to patients. In addition, improper cleaning in the kitchen (leading to floors, walls and ceilings that were not free from visible dust and soot) observed during the fourth sampling rounds, as well as the structural defects (such as holes and cracks in the wall) on the premises may serve as possible sources of airborne microbial contamination of food. *B. cereus* can cause food-borne illnesses with symptoms such as nausea, vomiting and diarrhea, and is known to be harmful to people with weakened immune systems [6]. Moreover, when samples were collected in the female ward prep room, *B. cereus* was present and its presence may be due to aerosols from the kitchen area travelling from one room to another since the kitchen area is located close to the male ward, or alternatively by means of the clothing of hospital personnel (Tables 1, 2 and 3).

**Table 1:** Bacterial characterisation: kitchen area

**Origin**	**Species identification (Gram (+) bacteria) using MALDI-TOF MS**	**Species identification (Gram (+) bacteria) using API**	**Source**	**Health effects**	**References**
**Kitchen area**	*Bacillus cereus* 994000168 LBK	*Bacillus cereus*	Soil	Food-borne illness causing severe nausea, vomiting and diarrhea	[28]
	*Bacillus cereus* 4080 LBK				
	*Bacillus cereus* DSM 31 T DSM				

**Table 2:** Bacterial characterisation: female wards

**Origin**	**Species identification (Gram (+) bacteria) using MALDI-TOF MS**	**Species identification (Gram (+) bacteria) using API**	**Source**	**Health effects**	**References**
**Female ward corridor**	*Micrococcus luteus* N203 CPB	*Micrococcus* spp.	Soil, dust, water and air	Skin infection	[29,30]
	*Staphylococcus lugdunensis* DSM 4805 DSM				
**Female ward Room 40**	*Corynebacterium afermentans* spp. *afermentans* 72_D4_coll ISB	*Corynebacterium* spp.	Soil, water, plant, and food products	Causes diphtheria	[31]
	*Corynebacterium glaucum* DSM 44530 T DSM				
**Female ward preparation room**	*Bacillus cereus* 4080 LBK		Soil	Food-borne illness causing severe nausea, vomiting and diarrhea	[30]
	*Bacillus cereus* 994000168 LBK				
	*Arthrobacter* spp. DSM 20125_DSM				
**Diabetic female ward**	*Kocuria rosea* IMET 11363 T HKJ	*Micrococcus* spp. *Staphylococcus* spp.	Soil, alkaline waste water.		[32]
	*Arthrobacter oxydans* DSM 20119 T DSM				
	*Arthrobacter tumbae* DSM 16406 T DSM				

**Table 3:** Bacterial characterization: male wards

**Origin**	**Species identification (Gram (+) bacteria) using MALDI-TOF MS**	**Species identification (Gram (+) bacteria) using API**	**Source**	**Health effects**	**References**
**Male ward corridor**	*Arthrobacter oxydans* IMET 10684 T HKJ	*Micrococcus* spp.	Soil, alkaline waste water	Causes severe irritation to humans	[28,32]
	*Arthrobacter* spp. B514 DSM 20389 UFL				
	*Arthrobacter oxydans* DSM 20119 T DSM				
**Male ward Room 3**	*Micrococcus luteus* IMET 11249 HKJ	*Micrococcus* spp.	Soil, dust, water and air	Skin infection	[28]
	*Micrococcus luteus* BK_01140_09 ERL				
	*Micrococcus luteus* N203 CPB				
**Male ward Room 4**	*Micrococcus luteus* IMET 11249 HKJ	*Micrococcus* spp.	Soil, dust, water and air	Skin infection	[28]
	*Micrococcus luteus* N203 CPB				
**Male ward Room 5**	*Micrococcus luteus* IMET 11249 HKJ	*Micrococcus* spp.	Soil, dust, water and air	Skin infection	[28]
	*Micrococcus luteus* N203 CPB				
	*Micrococcus luteus* BK_01140_09 ERL				
**Male ward TB room**	*Staphylococcus hominis* Mb18788_1 CHB	*Staphylococcus* spp. and *Micrococcus* spp.	Exterior of human ear and animals	Causes human skin infections and food poisoning	[28,29]
	*Staphylococcus hominis* ssp. *hominis* DSM 20328 T DSM				
	*Staphylococcus hominis* spp. *novobiosepticus* DSM 15614 T DSM				

Following the genus *Bacillus*, *Kocuria* (previously described as *Micrococcus*) and *Staphylococcus* were predominant genera found in eight different wards (Table 2 and 3). *S. aureus* was mainly found in the female ward, and similar results were also observed in other studies [19] where this bacterium was predominantly found in the female wards. The predominance of *S. aureus* could be due to the fact that female patients tend to get more visitors than male patients – an observation that was made in the current study. Visitors and multiple patients per room have been shown to influence the microbial rate of airborne bacteria in indoor air in hospitals [19]. *S. aureus* could increase in such cases since it is part of the skin’s microbial flora [33] that can be shed from the human skin.

*Kocuria* and *Staphylococcus* are Gram-positive bacteria isolated from soil, dust, water and air causing food poisoning, urinary tract infection and human skin infections such as folliculitis, boils, impetigo, and cellulitis [5,29]. Members of the genera *Staphylococcus* and *Kocuria* are characterized as catalase-positive Gram-positive cocci and both are hospital-acquired pathogens belonging to the family Micrococcaceae. Their presence in health-care settings should not be overlooked as it places patients staying in the hospital at high risk of contracting these opportunistic pathogens [34]. The presence of *S. aureus* may be due to aerosols dispersed by food handlers as well as by extensive handling by nurses, during visits to patients and from changing of bed linen. Moreover, food handlers carrying enterotoxigenic staphylococci in their nostrils (or skins) and handling cooked food products were implicated in a staphylococcal food poisoning outbreak [5,35]. *Kocuria*, considered a natural part of the skin’s microbial flora [33], is also an opportunistic pathogen causing bacteremia, septic shock, septic arthritis, and endocarditis especially to immuno-compromised patients such as HIV positive patients. *Kocuria* has also been reported in patients undergoing peripheral blood stem cell transplantation in hospitals [33]. Possible sources of these bacteria may be personnel, visitors and multiple patients per room [32].

The female ward preparation room, diabetic female wards and male ward corridor (Tables 2 and 3) had *Arthrobacter* as predominant bacteria found. In the current study, *Arthrobacter oxydans* and *Micrococcus luteus* were identified as predominant bacteria in both male and female wards and, according to the phylogenetic tree based on 16S rRNA gene sequences analysis [32], *Micrococcus luteus* is closely related to *Arthrobacter oxydans*; they have the same characteristics [32], with both of them usually originating from humans and soil. While in other studies *A. oxydans* was reported in clinical samples [32], a limitation in the current study was that no attempts were made to correlate air samples with clinical samples since this was the first time air sampling was conducted at this hospital. The current results do however emphasize the importance of using sensitive and rapid identification techniques such as the MALDI TOF MS as the identity of these microorganisms may easily be confused when using conventional techniques such as API. Even though molecular techniques may be used to identify microorganisms, these techniques are often time-consuming in comparison to the MALDI-TOF MS.

Fungi were isolated and identified in both male and female wards. Results obtained (Table 4) indicated that *Candida*, *Aureobasidium*, *Phoma exigua*, *Agromyces* and *Penicillium* were the predominant yeasts and moulds identified, known to cause fungal infections to patients. *Candida* species were identified mainly from samples collected in the kitchen area, diabetic female wards, male ward Room 3, male ward Room 5 and male ward TB ward. The presence of this fungus in the TB and diabetic wards is disturbing because it can result in candidiasis especially to vulnerable patients suffering from diabetes mellitus, HIV/AIDS and cancer [9]. The spread of these fungal hospital acquired infection-causing airborne contaminants in the indoor environment at hospitals may be attributable to open windows, inadequate air filtration systems or contamination of damaged surfaces such as ceilings, holes, and cracks.

**Table 4:** Fungal characterisation: kitchen, female and male wards

**Origin**	**Species identification using MALDI-TOF MS**	**Species identification using API**	**Source**	**Health effects**	**References**
**Kitchen area**	*Candida kefyr* [anamorph] (Kluyveromyces *marxianus* spp. *marxianus* [teleomorph]) CBS 834	*Candida* spp.	Plant debris, soil, wood, textiles, indoor air environment	Causes pneumonia, keratomycosis, pulmonary mycosis with sepsis eumycotic dermatitis, peritonitis, etc.	[36,37]
	*Aureobasidium pullulans* 16419 CBS BS				
	*Aureobasidium pullulans* 12235 CBS				
**Diabetic female ward**	*Candida krusei* [anamorph] (*Issatchenkia orientalis*[teleomorph]) ATCC 14243 THL	*Candida* spp.	Plant debris, soil, water, wood, textiles, food products, indoor and outdoor air environment	Candidiasis with fungal infections of the skin, mucous membranes and internal organs	[36,37]
	*Phoma exigua* spp. *exigua* CBS 431_74 CBS				
	*Candida robustad* [anamorph] (*Saccharomyces cerevisiae* [teleomorph]) INVSc1 BRL				
**Male ward Room 3**	*Candida glabrata* ATCC 2001 T THL	*Candida* spp.	Plant debris, soil, water, wood, textiles, food products, indoor and outdoor air	Candidiasis with fungal infections of the skin, mucous membranes and internal organs	[36,37]
**Male ward Room 5**	*Agromyces rhizospherae* HKI 302_DSM 14597 T HKJ	*Agromyces rhizospherae*	Plant debris, soil, wood, textiles, and indoor air environment	Causes pneumonia, keratomycosis, pulmonary mycosis with sepsis eumycotic dermatitis, peritonitis, etc.	[36,37]
	*Candida parapsilosis* ATCC 22019 THL				
**Male ward TB room**	*Aureobasidium pullulans* 16420 CBS	*Penicillium* spp.	Plant debris, soil, wood, food products, textiles, and indoor air environment	Causes pneumonia, keratomycosis, peritonitis, etc. hypersensitivity pneumonitis, asthma, allergic alveolitis	[36,37]
	*Penicillium* spp. IsolateS2 HED				
	*Candida orthopsilosis* P3118_8_37 HAC				

Fungal spores usually accumulate when dust particles enter the patient room via personnel’s clothing. Another element that encourages the proliferation of airborne fungi can be moisture as fungi proliferates in moist environments [19]. In addition, medical interventions such as insertion of catheters, fluids and nutrients inhalation, and wounds, as well as prolonged hospitalisation, have been reported as possible causes of candidiasis leading to infections of the skin, mucous membranes and internal organs [38,39]. Moreover, Pfaller et al. [40] report that candidiasis is the most common cause of bloodstream infections, which are mostly acquired during the hospital stay. Studies done by Miller and colleagues in 2001 showed that the cost of invasive candidiasis was approaching $1 billion per year [22,41]. Various studies cited indicate that the spread of *Candida* takes place via the contact route; however, results from the current study indicate that a possibility exists that the spread of this fungus may also be via the aerial route. These results may have serious implications for health-care settings; however, future studies will have to be done to confirm the spread of this fungus via the aerial route. Air samples will have to be correlated with clinical samples in future studies. Furthermore these findings indicate a need to control hospital acquired pathogens especially if these pathogens may be airborne.

In male ward Room 4, male ward TB room and the kitchen area, the yeast identified was *Aureobasidium pullulans. A. pullulans* is found in soil, water, air and limestone; it causes fungal infections that are more likely to occur in immuno-suppressed patients with symptoms such as pneumonia, asthma, dermatitis, keratitis and respiratory system irritation. The fungus has been implicated in an HAI case by Hermenides-Nijhof [42]. Reported possible sources of infection may be surgical procedures that provide several entry sites for the organism to survive; traumatic injuries such as road accidents leading to contact with the soil (rich reservoir of microorganisms) and accidental contamination of the temporary catheters used during the hospital stay [43].

*Penicillium* was also identified in the TB ward. This genus is found in the soil, air, dust and on salted food products such as cheese, meat, seeds, bread, fruits, etc. causing food spoilage [40,44,45]. The presence of *Penicillium* in the TB ward is concerning as inhalation can lead to hypersensitivity pneumonitis, asthma, and allergic alveolitis in susceptible individuals [44]. Results obtained show a need for constant air monitoring as well as identifying the source of this fungus as it has serious health implications in this ward. Follow up studies shall be conducted to confirm the survival of these fungi in the TB ward at this hospital as it has a UV irradiation system. *Phoma exigua*, commonly found in soil, was identified in the diabetic female ward as a predominant organism [46,47]. Infection by *Phoma exigua* causes phaeomycotic cysts especially to vulnerable patients, leading to symptoms such as fever, painful joints, and tumors. Previous studies have reported the presence of this fungus in a diabetic ward [48]. Results from the current study and other studies indicate that diabetic patients may be the source of this organism as it was isolated in the diabetic ward only. No attempts were made in the current study to verify this claim since it was the first time air sampling was conducted. However, due to the significance of their impact air samples will be correlated with clinical samples in future studies.

## Conclusions

This is a first report on the presence of bio-aerosols at a district hospital in South Africa. Even though this government hospital is old (built in 1892) microbial counts obtained in this study were generally low when correlated with other results obtained by Qudiesat et al. [19] and Nkhebenyane [5]. Higher counts were observed during passive sampling when compared with active sampling an indication that microbial contaminants may settle on hospital surfaces possibly resulting in acquired infections. However, because a preliminary walk through was not conducted prior sampling, factors that affected bio-aerosol recovery were not investigated and this will be considered in future studies. Observations made during sampling rounds found that, floors, walls, painted surfaces and ceilings were not free from visible dust, soot, holes and cracks. This was of concern as it could lead to an increase in microbial contaminants. Lack of limitations on the time duration of visits at this hospital may also increase the proliferation of airborne contaminants. Other factors that influenced the levels of indoor air microorganisms especially in the kitchen areas were poor and deficient hygiene conditions, a low degree of cleanliness and minimal disinfection procedures used against airborne contaminants. The presence of opportunist pathogens was of concern as these may lead to HAI. Therefore, to reduce contamination in this hospital, frequent air monitoring and educational training for food handlers is needed. Moreover, future studies also need to be done to determine if the airborne bacteria found on hospital premises are also present in clinical samples and not resistant to antibiotics. Additionally, results obtained in this study indicate the MALDI TOF MS as the best technique for the analysis and fingerprinting of unknown airborne microbes especially bacteria in healthcare settings.

## Competing interests

The authors declare that they have no competing interests.

## Authors’ contributions

GG conceived the study and have made substantial contribution to acquisition, analysis and interpretation of data. NJ, K and JFR equally have contributed substantially to conception and design and provided important review of the manuscript for significant intellectual content. NJ also gave final approval of the article to be published. All authors read and approved the final manuscript.
